# Immunohistochemistry – Microarray Analysis of Patients with Peritoneal Metastases of Appendiceal or Colorectal Origin

**DOI:** 10.3389/fsurg.2014.00050

**Published:** 2015-01-05

**Authors:** Danielle E. Green, Thejus T. Jayakrishnan, Michael Hwang, Sam G. Pappas, T. Clark Gamblin, Kiran K. Turaga

**Affiliations:** ^1^Division of Surgical Oncology, Department of Surgery, Medical College of Wisconsin, Milwaukee, WI, USA; ^2^Division of Surgical Oncology, Department of Surgery, Loyola University Medical Center, Maywood, IL, USA

**Keywords:** colorectal neoplasms, appendiceal neoplasms, peritoneal neoplasms, microarray analysis, immunohistochemistry, DNA mutational analysis

## Abstract

**Background:** The value of immunohistochemistry (IHC)-microarray analysis of pathological specimens in the management of patients is controversial, although preliminary data suggest potential benefit. We describe the characteristics of patients undergoing a commercially available IHC-microarray method in patients with peritoneal metastases (PM) and the feasibility of this technique in this population.

**Methods:** We retrospectively analyzed consecutive patients with pathologically confirmed PM from appendiceal or colorectal primary who underwent Caris Molecular Intelligence^™^ testing. IHC, microarray, FISH, and mutational analysis were included and stratified by Peritoneal Carcinomatosis Index (PCI) score, histology, and treatment characteristics. Statistical analysis was performed using non-parametric tests.

**Results:** Our study included 5 patients with appendiceal and 11 with colorectal PM. The median age of patients was 51 (IQR 39–65) years, with 11 (68%) female. The median PCI score of the patients was 17 (IQR 10–25). Hyperthermic intra-peritoneal chemoperfusion was performed in 4 (80%) patients with appendiceal primary tumors and 4 (36%) with colorectal primary. KRAS mutations were encountered in 40% of appendiceal vs. 30% colorectal tumors, while BRAF mutations were seen in 40% of colorectal PM and none of the patients with appendiceal PM (*p* = 0.06). IHC biomarker expression was not significantly different between the two primaries. Sufficient tumor for microarray analysis was found in 44% (*n* = 7) patients, which was not associated with previous use of chemotherapy (*p* > 0.20 for 5-FU/LV, Irinotecan and Oxaliplatin).

**Conclusion:** In a small sample of patients with PM, the feasibility and results of IHC-microarray staining based on a commercially available test is reported. The apparent high incidence of the BRAF mutation in patients with PM may potentially offer opportunities for novel therapeutics and suggest that IHC-microarray is a method that can be used in this population.

## Introduction

Predictive and prognostic biomarkers have been studied in different cancers with the aim of developing personalized medicine for patients (1, 2). The role of immunohistochemistry (IHC)-microarray analysis of pathological specimens in this paradigm is currently being investigated, and preliminary data do suggest potential utility (3, 4). Few studies have examined the value of gene expression and molecular profiling of specimens in the setting of peritoneal metastases (PM) (5–7). However, the role of IHC-microarray analysis of specimens from PM of appendiceal or colorectal origin is not well elucidated.

Gene expression profiling has pointed toward phenotypic clustering with distinct survival outcomes for patients with PM (8). Vascular endothelial growth factor (VEGF) has been suggested as a potential prognostic marker for patients with PM from mucinous adenocarcinoma originating in the appendix or colon (9, 10). In colorectal cancer, there is evidence to support the association of malignant transformation and peritoneal dissemination with BRAF/KRAS mutations (11–14). Genes in the RAF family encode kinases regulated by the Ras pathway and mediate cell responses to growth signals. As such, BRAF is seen as a gene downstream from KRAS. Interestingly, BRAF and KRAS mutations have been observed to be mutually exclusive even though they exert equal effects on tumorigenesis (15).

Also important in colorectal cancer, cyclooxygenase-2 (COX-2) is an enzyme that is upregulated in colorectal cancer (16, 17). COX-2 inhibitors, like aspirin, have a protective effect on the development of colorectal cancer, and have been shown to improve survival in COX-2 overexpressing tumors (18, 19). The therapeutic value of COX-2 inhibition in appendiceal tumors is not clearly defined although studies have demonstrated increased expression (20).

Demonstration of the clinical utility of molecular profiling in guiding successful systemic therapy have resulted in a surge in the use of commercial molecular profiling services (21). The purpose of this study was to assess the feasibility of routine IHC-microarray and mutational analysis of patients with PM of appendiceal and colorectal primaries using a commercially available molecular profiling service. This is important in the context of mucinous contents of these tumors that could impact the sampling and analysis.

## Materials and Methods

### Subjects

A retrospective chart review with Institutional Review Board (IRB) approval was performed to identify all patients that had appendiceal or colorectal primaries with PM that also had tissue biopsies for molecular profiling (Caris Molecular Intelligence^™^) sent to Caris Life Sciences lab (Phoenix, AZ, USA), which is a central Clinical Laboratory Improvement Amendments-certified laboratory. Additional demographics, histology, surgery, and treatment details were collected from medical records. Resultant biomarker data were then stratified by Peritoneal Carcinomatosis Index (PCI), histology, and treatment characteristics. All patients were treated at the regional cancer therapy program at the Medical College of Wisconsin (MCW) between April 2008 and June 2012. Patients with insufficient tissue for molecular profiling to be performed or having primaries other than the colon or appendix were excluded from the study.

### Patient samples

Our standard practice for patients with high grade appendiceal adenocarcinoma or colorectal carcinomatosis is to evaluate the patients with a staging laparoscopy after four to six cycles of systemic chemotherapy. Biopsies for pathological and biochemical analysis were obtained during laparoscopy and shipped to Caris lab using standard protocol aimed to provide theranostic information (21). Sufficiency of the sample for biochemical analysis (Caris Molecular Intelligence^™^) was determined at the lab. Primary antibodies used for IHC are COX-2 (SP21), ERCC1 (8F1), PTEN (6H2.1), SPARC monoclonal (122511), SPARC polyclonal (polyclonal), TOP2A (3F6), TOPO1 (1D6), TS (TS106/4H4B1), and RRM1 (polyclonal). Avidin-biotin systems and polymer-based, biotin-free systems were used for detection of the antibodies. The chromogenic reporter, 3,3′-diaminobenzidine (DAB), was used to allow for a colorimetric visualization of the antibody yielding a brown stain that can be analyzed with a light microscope. The intensity of the staining was scored as 0, 1+, 2+, or 3+, and the percentage of staining cells was scored as 0–100% by a board-certified pathologist. Thresholds to determine patient benefit to therapeutic agents included the following: negative TS, staining smaller than or equal to 1+, 25%; negative ERCC1, percentage of staining smaller than or equal to 25%; high TOP2A, percentage of staining >10%. DNA microarray was performed on total RNA extracted from tumor tissue and was converted to cDNA. This cDNA sample was then subjected to a whole genome microarray analysis using Illumina cDNA-mediated annealing, selection, extension, and ligation (DASL) process with the HumanHT-12 v4 beadChip (Illumina Inc., San Diego, CA, USA). After direct hybridization and scanning of the bead array, the expression of a subset of 88 transcripts were then compared to tissue-specific normal control pools and the statistical significance of the difference between the patient tumor sample and control is determined. Direct sequence analysis was performed on genomic DNA isolated from a tumor sample using custom primers designed to flank, amplify and sequence codons 12 and 13 in exon 2 and codon 61 in exon 3 of the KRAS gene, and codons 464–469 of exon 11 and codon 600 of exon 15 in the BRAF gene.

### Treatment

Following laparoscopic evaluation, patients were classified as extensive disease for exclusion from Cytoreductive Surgery with hyperthermic intra-peritoneal chemoperfusion (CRS + HIPEC) for PCI scores higher than 11 for gastric cancer, and 19 for colorectal and non-gastric primaries. After cytoreduction, HIPEC was performed using the closed technique and dosing of therapy was based on standard published consensus guidelines (22).

### Statistical analysis

Statistical calculations were performed using STATA software version 12.1 (StataCorp, TX, USA). Non-parametric methods of Kruskal Wallis and chi-square tests were employed. Survival outcomes were explored using Cox proportional hazards model. Alpha was fixed at 0.05 for statistical significance.

## Results

The baseline characteristics and survival of the subjects are described in Table 1. Of the 16 patients, 11 had colon primaries and 5 had appendix primaries. Biopsies were sufficient in size for IHC to be performed on 16 patient samples vs. 7 for microarray. Patients with colon cancer were twice as likely to undergo neoadjuvant chemotherapy vs. those with appendiceal cancer that were twice as likely to undergo HIPEC upfront.

**Table 1:** **Baseline characteristics and survival of colon and appendix cancer patients included in the study**.

	All patients (*n* = 16)	Colon (*n* = 11)	Appendix (*n* = 5)	*p*-value
Age (median and range in years)	51 (42–60)	51 (38– 72)	51 (40– 56)	0.42
Female	11 (69%)	8 (73%)	3 (60%)	1.00
AJCC6 T-stage T4	11 (69%)	8 (73%)	3 (60%)	1.00
Neoadjuvant chemotherapy	11 (69%)	9 (82%)	2 (40%)	0.24
PCI score (median and range)	17 (13–26)	17 (8– 27)	17 (12– 20)	0.82
CRS + HIPEC	8 (50%)	4 (36%)	4 (80%)	0.28
Median survival (months)	–	30	60	0.50

*AJCC, American Joint Committee on Cancer; PCI, Peritoneal Carcinomatosis Index; CRS + HIPEC, cytoreductive surgery with hyperthermic intra-peritoneal chemoperfusion*.

### Immunohistochemistry (IHC)

Immunohistochemistry results are described in Table 2. Differences in percent staining or staining intensity were not significantly different between the two cancer primaries (*p*-value = 0.20). Of note, COX-2 was not differently expressed between the two groups with 80% staining in colorectal vs. 100% in appendiceal (*p*-value = 0.52).

**Table 2:** **Immunohistochemistry (IHC) findings stratified by histological subtype**.

Biomarker (IHC)	Colon (*N* = 11)	Appendix (*N* = 5)	*p*-value
COX-2	80%	100%	0.52
ERCC1	100%	100%	1.00
PTEN	100%	100%	1.00
SPARC	36%	20%	1.00
TOP2A	100%	80%	0.31
TOPO1	100%	100%	1.00
TS	82%	80%	1.00

*COX-2, cyclooxygenase-2; ERCC1, excision repair cross-complementing rodent repair deficiency, complementation group 1; PTEN, phosphatase and tensin homolog; SPARC, secreted protein acidic cysteine-rich; TOP2A, topoisomerase (DNA) 2 alpha; TOPO1, topoisomerase (DNA) 1; TS, thymidylate synthase*.

### Mutational analysis

**Figure 1:**
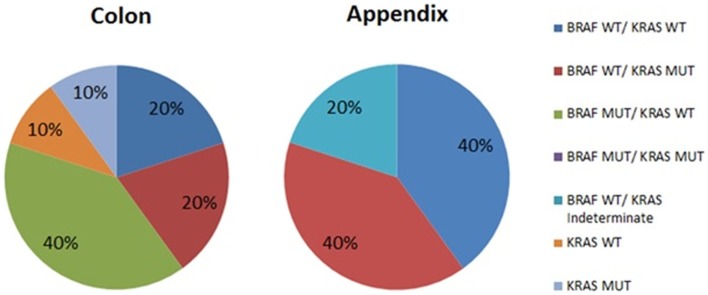
**Mutational analysis of peritoneal metastases from colon and appendix primaries**. WT, wild type; MUT, mutated.

The results of mutational analysis (*n* = 15) are represented in Figure 1. Patients with appendiceal primary were all BRAF wild type, but were split between KRAS wild type (40%) and KRAS mutant (40%). The analysis was reported as “indeterminate KRAS” for 1 patient (20%). Patients with colon primary had more variation in their mutations with the most common mutation being BRAF mutated with KRAS wild type (40%) vs. 20% with BRAF wild type–KRAS wild type vs. 20% BRAF wild type–KRAS mutant. Of two patients who did not have BRAF mutation analysis performed, one was KRAS mutant.

### Survival

The results of survival analysis are described in Table 3. When biomarkers of interest were compared with survival data, COX-2 was the only biomarker that significantly impacted survival (expression associated with a hazards ratio of 0.06, *p*-value = 0.02). SPARC (Poly) expression also showed trends toward survival benefit (hazards ratio 0.15, *p*-value = 0.08).

**Table 3:** **Unadjusted survival analysis stratified by biomarkers identified in immunohistochemistry (IHC)**.

Biomarker	Hazards ratio (95% CI)	*p*-value
COX-2	0.06 (0.005–0.71)	0.02
RRM1	0.26 (0.04–1.57)	0.14
SPARC (Poly)	0.15 (0.02–1.3)	0.08
TS	1.00	1.00

*COX-2, cyclooxygenase-2; RRM1, ribonucleotide reductase M1; SPARC, secreted protein acidic cysteine-rich; TS, thymidylate synthase*.

## Discussion

Use of a commercial molecular profiling service can yield significant information toward the understanding of biology and treatment response of PM in patients with appendiceal or colorectal primaries. While concerns of mucinous disease interfering with the test exist, we found that majority of the patients were able to successfully undergo IHC evaluation of their samples, although microarray could not be performed in some.

Previous studies have revealed that the phenotypes could be clustered based on the gene expression profile. Levine et al. reported gene expression profiling in 41 samples of patients with PM of appendiceal and colorectal origin and showed that the two groups were genomically different with impact on survival (8). They identified 3 different phenotype clusters with the following results: high risk colorectal cancer group with worst prognosis (0% 5 years OS), high- and low-risk appendiceal subgroups (25 and 70% 10-year OS, respectively) (8).

The important role of BRAF/KRAS mutations in the tumorogenesis pathway has been well elucidated (11, 23). Chan et al. showed higher prevalence of BRAF or KRAS mutations (90%) in serrated polyps with dysplasia compared to polyps without dysplasia (54%), thereby suggesting a role in malignant transformation (23). Some authors failed to show increased prevalence of BRAF mutations in dysplastic specimens compared to non-dysplastic serrated polyps and have argued that these mutations were associated with serrated morphology rather than malignant transformation (11, 24). Mutations in KRAS have been associated with metastatic colorectal cancer including peritoneal dissemination with implications for therapy (12, 25–27). Mutations in BRAF are involved in sporadic colorectal tumorigenesis with proclivity to develop PM, as well as poor survival outcomes compared to those with KRAS mutations (13, 14).

In attempting to understand the profile of these tumors, the high proportion of BRAF mutated tumors in the colorectal primary patients with PM is remarkable. Increasing evidence suggests that the phenotype of BRAF mutated tumors presents with PM, and aggressive biology, which is what we found in this small cohort of patients (13, 14). Distinct in their genetic code, appendiceal tumors did not express BRAF mutations, which confirm a distinct origin from their counterparts, as suggested by other authors as well (28, 29). Our study confirmed the high prevalence of KRAS mutations (40%) in patients with appendiceal primaries, which are similar to previous studies in which one found 45% with KRAS mutations and another 91% with KRAS mutations among those patients with appendiceal primaries with PM (30). We did not encounter patients that exhibited both BRAF and KRAS mutations simultaneously, which is also consistent with previous reports (15).

There is a possible survival benefit for patients with COX-2 expression especially in the setting of PM, which may lend credence to the use of aspirin or other NSAIDs in the management of patients with peritoneal carcinomatosis. Although previous studies have identified the benefit of COX inhibitors in the treatment and prevention of colorectal cancer, these data have not included disease with PM (18, 19). Molecular studies on appendiceal adenocarcinoma also revealed increased expression of COX-2 but failed to identify a prognostic value or therapeutic benefit with targeted therapy (20). In light of this, more data would need to be collected to analyze the effect COX inhibitors on PM with high COX-2 expression.

The purpose of molecular profiling is to guide therapies. Appendiceal cancer is a rare primary and molecular profiling of this histological subtype could be extremely useful when dealing with high grade disease to determine potential molecular targets. We were limited in our analysis due to our small sample size of 16 and also the insufficient sample for more than half of our patients (56%) to have microarray analysis performed. In this study, microarray analysis was limited by the amount of tumor biopsied as well as by the mucinous content of the tumor. While this sample may be too small and therefore underpowered to detect potential targets for intervention, it does demonstrate the feasibility of this novel technique for patients with PM. It is noteworthy to point out that we were able capture variations in the expression of molecular markers and trends in survival benefit despite the small size.

## Conclusion

Immunohistochemistry and mutational analysis using commercially available molecular profiling testing is feasible for patients undergoing surgery for their PM, although mucin content might interfere with microarray analysis. BRAF mutations and COX-2 expression deserve further investigation in the prognostication and management of patients with colorectal and appendiceal primaries.

## Conflict of Interest Statement

Dr. Kiran K. Turaga has received honorarium from Castle Life Sciences. The other authors have no disclosures to make. Dr. Joanne Xiu from CARIS Life Sciences assisted with manuscript review to verify accuracy of the methods of the techniques used for IHC and microarray analysis. There was no financial conflict of interest between the company and the authors of the manuscript.

## Author Note

This study was presented at the Eighth International Symposium on Regional Cancer Therapies held February 16–18, 2013 in Indian Wells, California.
